# The fetal exposome: cellular and epigenetic programming of neonatal immune function

**DOI:** 10.3389/fimmu.2026.1896547

**Published:** 2026-08-10

**Authors:** William Dodwell, David Cousins, Elaine Boyle, Thillagavathie Pillay

**Affiliations:** 1 Division of Respiratory Science, School of Medical Sciences, University of Leicester, Leicester, United Kingdom; 2 College of Life Sciences, School of Healthcare, University of Leicester, Leicester, United Kingdom; 3 Leicester Neonatal Service, University Hospitals of Leicester NHS Trust, Leicester, United Kingdom; 4 Faculty of Science and Engineering, University of Wolverhampton, Wolverhampton, United Kingdom

**Keywords:** allergic disease, developmental programming, Developmental Origins of Health and Disease (DOHaD), epigenetics, fetal exposome, immune programming, maternal exposure, neonatal immunology

## Abstract

Fetal life represents a critical period in the development of the neonatal immune system. During this period of high epigenomic plasticity, environmental exposures, maternal health conditions and perinatal factors can have a lifelong impact on gene expression and function, influencing neonatal immune function and contributing to the later development of immune-mediated disease. We conducted a narrative review of human and translational evidence linking antenatal exposures with cellular and epigenetic regulation of neonatal immune function. Evidence was synthesised across maternal health, environmental and perinatal exposures, including the maternal microbiota as a potential exposome–epigenome interface, with a focus on immune-cell composition, cytokine and immunoglobulin profiles, functional immune responses and epigenetic mechanisms including DNA methylation, histone modification and microRNA regulation. Across diverse exposures, including maternal smoking, obesity, air pollution and heavy metals, antenatal factors were associated with altered neonatal immune-cell composition, dysregulated cytokine production and changes in innate and adaptive immune responses. Reported epigenetic alterations involved genes and pathways related to haematopoiesis, antigen presentation, cytokine regulation, immune-cell activation, metabolic signalling and inflammatory responses. The reviewed evidence suggests that the fetal exposome may shape neonatal immune development through epigenetic programming, with recurrent patterns including type 2 polarisation, impaired regulatory function and altered innate immune signalling. These effects may be particularly relevant in socially deprived populations, where multiple adverse exposures frequently cluster. However, evidence remains heterogeneous, and longitudinal studies integrating exposure assessment, multi-omic immune profiling and clinical follow-up are needed to define functional relevance and long-term disease risk.

## 1 Introduction

**Figure 1:**
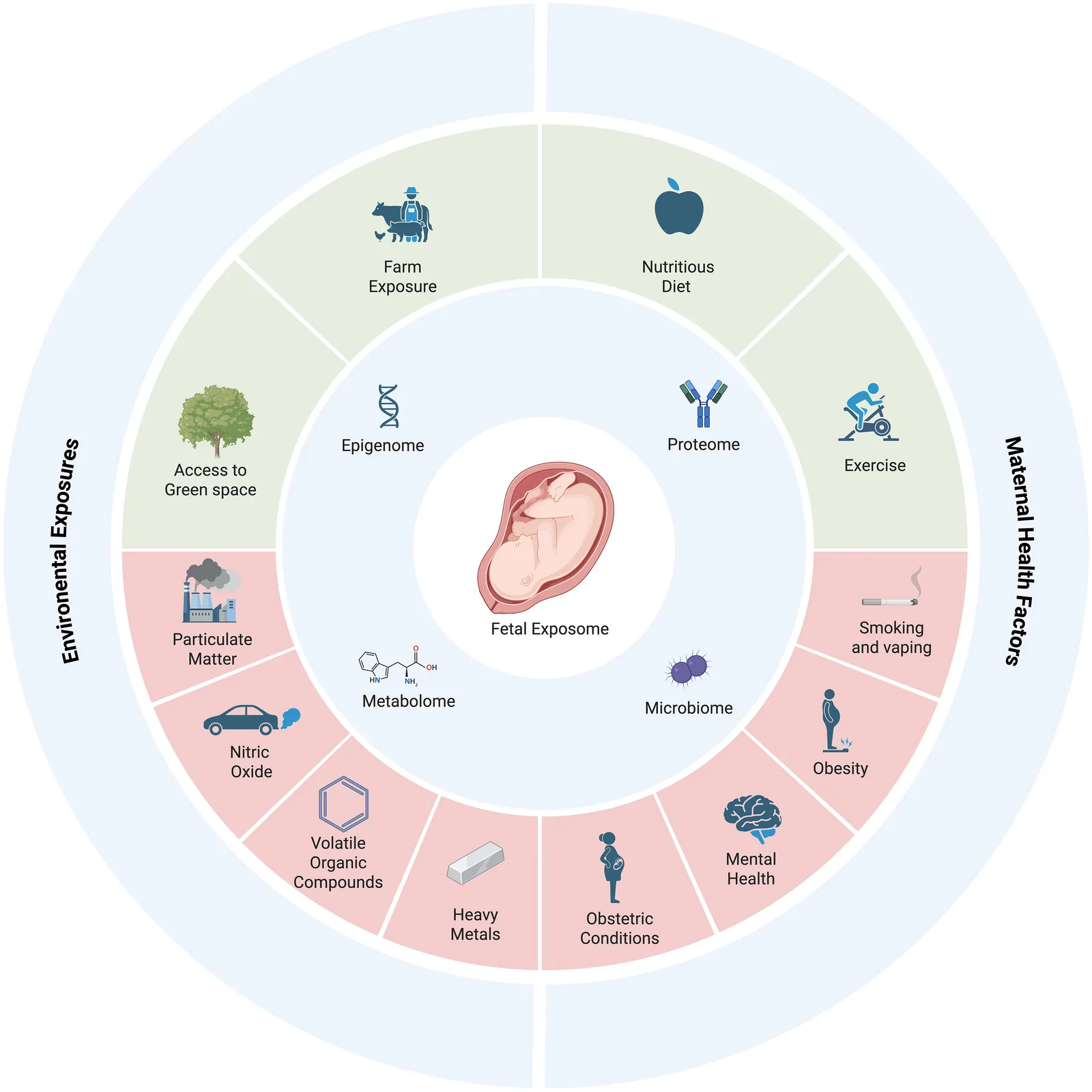
Schematic of the fetal exposome. The fetal exposome comprises the totality of maternal and environmental exposures during pregnancy that influence fetal immune function through effects in the epigenome, metabolome, proteome and microbiome. Exposures coloured red indicates negative overall impact on the fetal exposome, whereas green implies positive effect. Created in BioRender. Dodwell, W (2026). https://BioRender.com/i16y880.

Fetal life represents a critical period in the development of the immune system. During this period of high epigenomic plasticity, maternal health and environmental exposures can have a lifelong impact on gene expression and function (1). This can lead to lasting changes in both the composition and function of the neonatal immune system, a process known as developmental programming (2). This concept forms part of the Developmental Origins of Health and Disease (DOHaD) hypothesis, which describes how exposures during fetal and early-life development can shape later health and disease risk (3). The totality of these antenatal exposures constitutes the fetal exposome (**Figure 1**).

Antenatal exposures are important determinants of pregnancy, neonatal and childhood health. Cigarette smoke, air pollutants, and environmental chemicals, as well as maternal factors such as obesity and malnutrition, are associated with higher rates of stillbirth, preterm birth, sudden infant death syndrome and immune-mediated disorders (4–7).

Importantly, the fetal exposome is not equal across populations (8–14). Social deprivation amplifies vulnerability through the cumulative effects of multiple adverse environmental, nutritional and maternal health exposures in the same pregnancies. This is reflected in epidemiological studies showing strong associations between social deprivation and adverse pregnancy and childhood health outcomes. Within the United Kingdom, infant mortality rises by 10% for each decile decrease in Index of Multiple Deprivation (IMD) score (15). Similar disparities are observed across immune-mediated disorders, including asthma, atopic disease, and neonatal sepsis (16–20).

These observations raise an important question: whether antenatal exposures contribute to adverse neonatal and childhood outcomes through their impact on the development and function of the immune system. While the adverse health outcomes associated with the fetal exposome are increasingly well described in the literature, less is known about how these exposures shape neonatal immune function and the epigenetic mechanisms that underpin these effects (4–14, 21–23).

This narrative review therefore examines how key components of the fetal exposome influence neonatal immune development, with a focus on the epigenetic mechanisms that shape immune programming.

## 2 Epigenetic regulation of gene expression

A key mechanism of developmental programming occurs through the epigenetic regulation of gene expression. Epigenetic regulation alters the accessibility, stability or translation of genetic information without changing the underlying DNA sequence (24). During fetal life, these processes are particularly important because immune-cell lineages are emerging, expanding and acquiring functional specialisation during a period of exceptional epigenomic plasticity. Environmental exposures during this window may therefore alter not only transient inflammatory responses but also drive persistent changes in immune composition, signalling and immune tolerance (2).

**Figure 2:**
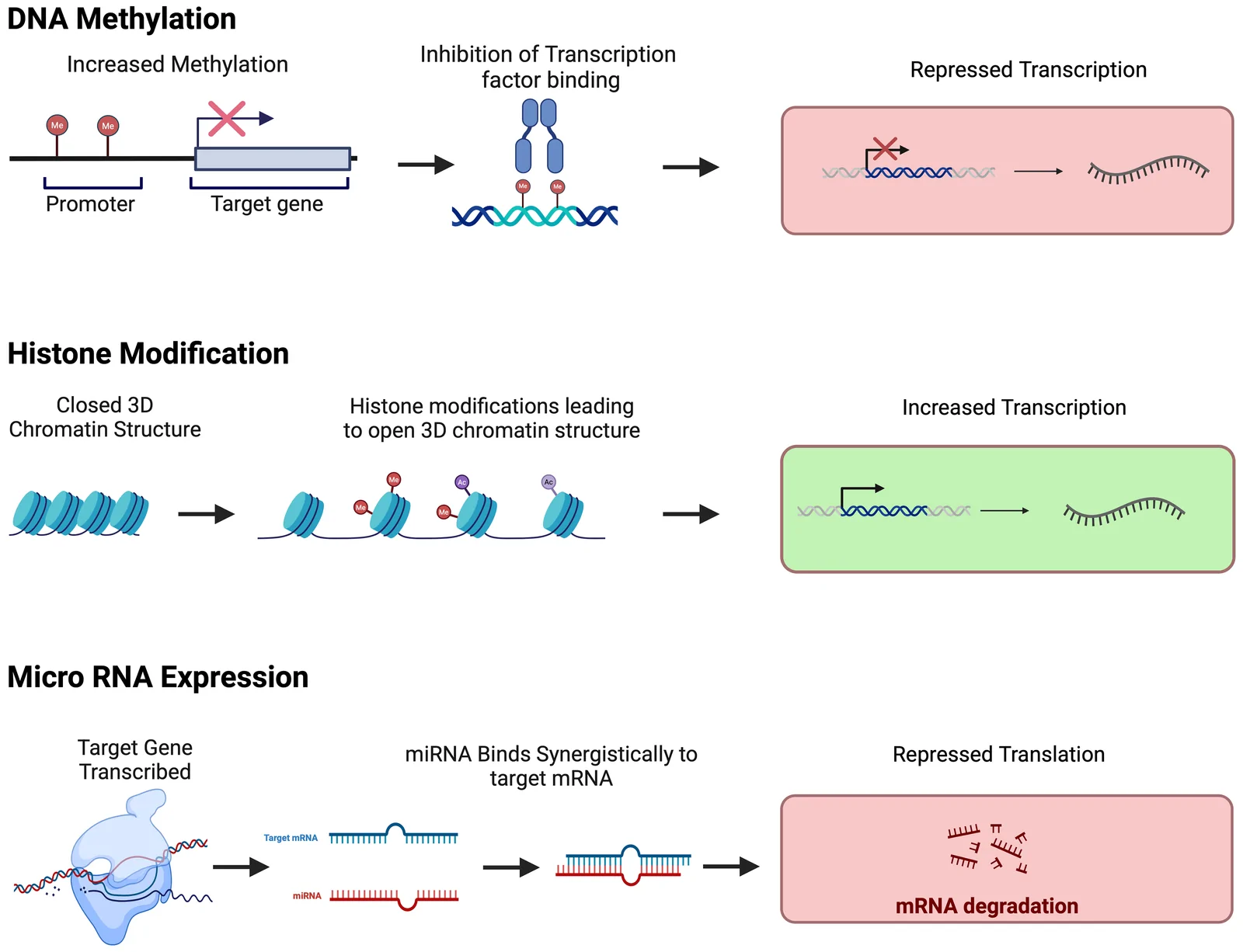
Epigenetic mechanisms. DNA methylation involves the addition of methyl groups to DNA. Promoter methylation can inhibit transcription-factor binding and downregulate gene expression, whereas gene-body methylation can have divergent effects; Histone modifications involve the addition of chemical groups to histone proteins which influence 3D chromatin structure, either opening it, making it easier for the binding of transcription factors, thereby promoting transcription or closing the structure and repressing transcription; MicroRNA expression influences gene expression through short non-coding RNAs which bind complementary target mRNAs, destabilising the mRNA and/or preventing translation. Created in BioRender. Dodwell, W (2026). https://BioRender.com/qya7bo2.

The principal mechanisms through which epigenetic regulation occurs include DNA methylation, histone modifications and microRNAs (**Figure 2**) (24–26). DNA methylation involves the addition or removal of methyl groups at regulatory regions of DNA and can thereby regulate gene expression by altering transcription-factor binding and chromatin accessibility (24, 25). Histone modifications involve the addition of chemical groups to histone proteins, influencing three-dimensional chromatin structure and determining whether chromatin is open and transcriptionally accessible or closed and transcriptionally repressed (24). MicroRNAs are short non-coding RNAs that bind complementary target mRNAs, destabilising the mRNA, thereby preventing translation (26).

External exposures do not generally modify the epigenome directly; instead, their effects are mediated through upstream signalling pathways in maternal, placental and fetal cells (27, 28). These pathways can alter transcription-factor activity and binding, leading to the recruitment of chromatin-remodelling complexes, histone-modifying enzymes and DNA methyltransferases (24). Through these mechanisms, environmental signals lead to coordinated changes across the fetal epigenome, thereby regulating gene expression in developing immune cells (2, 24).

## 3 Components of the fetal exposome

### 3.1 Maternal health

#### 3.1.1 Smoking

Antenatal exposure to cigarette smoke is strongly linked with adverse perinatal and neonatal outcomes, including stillbirth and neonatal mortality, as well as increased risk of immune-mediated disorders such as neonatal sepsis, asthma and allergic rhinitis (4, 6, 29). Smoke exposure influences fetal immune development through multiple, overlapping mechanisms, including placental dysfunction, oxidative stress, direct fetal toxicant exposure, xenobiotic-response signalling, altered immune signalling and epigenetic regulation (**Tables 1**, **2**). In the placenta, smoke exposure is associated with morphological changes, with reduced vascularisation and increased oxidative stress leading to impaired nutrient and oxygen transfer, while epigenetic studies have identified the differential regulation of multiple genes involved in immune function, haematopoiesis and xenobiotic metabolism (30–34).

**Table 1:** The impact of antenatal smoking exposure on neonatal immune function.

Characteristic	Impact		Ref
Cord Blood Immune Cell Frequencies	↓Nϕ ↓Mono ↓Basophil ↓DCs ↓T_H_ ↓T_R_		(61, 229–232)
Cytokine Profile	↓IL-7 ↑IL-8		(40, 233)
Immunoglobulin Profile	*↑tIgE ↑tIgG ↑tIgM ↑IgG-1 ↑IgG-3 ↑sIgA		(35–39, 234–241)
CBMC Characteristics	↓NK cytotoxic activity ↓Viability ↑Apoptosis ↑Proliferative responses		(49, 50)
Cytokine Response in Stimulated CBMCs	**Allergenic Stimulation ** HDM OVA **Mitogenic Stimulation ** PHA ConA TLR 2 TLR3/TLR4 TLR7/TLR8 TLR9 PMA/Ionomycin ConA/PMA	↑IL-13 ↑IL-13, ↑IL-5 ↓IFN-γ RNA ↓IFN-γ RNA ↓IL-6 ↓TNF-α ↓TNF-α ↑IFN-α and ↑TNF-α ↓IL-6 ↓IL-5 ↓IFN-γ	(40, 41, 48)
Markers of Oxidative stress	↑ROS		(50)
CBMC *in-vitro* Nicotine Exposure	↑TNF-α ↑IL-6 ↑TLR-2 ↑CD11A ↑CXCR4		(242)

Nϕ, Neutrophils; Mono, Monocytes; DC, Dendritic Cell; T_R_, Regulatory T cell; IL, Interleukin; Ig, Immunoglobulin; TIg, Total immunoglobulin; sIg, Secretory immunoglobulin; IFN, Interferon; TNF-α, Tumour necrosis factor-a; ROS, Reactive oxygen species; HDM, House Dust mite antigen; OVA, Ovalbumin; PHA, Phytohaemagglutinin; ConA, Concanavalin A; PMA, Phorbol myristate acetate; TLR, Toll-like receptor; CD11A, Integrin alpha L; CXCR4, chemokine receptor type 4.

Bold text indicates the type of stimulation used in stimulated CBMC experiments: allergenic stimulation or mitogenic stimulation.

↓, decreased; ↑, increased; *, see comment.

**Table 2:** Immune-relevant differentially methylated genes associated with antenatal smoking exposure.

Gene	Immune pathway implicated	Methylation change
AHRR *Aryl-hydrocarbon receptor repressor*	AHR signalling, xenobiotic response, immune-cell differentiation and inflammatory regulation	Hypomethylated
GFI1 *Growth factor independent 1 transcriptional repressor*	Granulocyte and lymphocyte haematopoiesis	Hypomethylated
MYO1G *Myosin IG*	Lymphocyte adhesion, migration and immune-cell trafficking	Hypermethylated
IL-10	Anti-inflammatory cytokine signalling and immune regulation	Hypermethylated
FOXP3	Regulatory T-cell differentiation and immunosuppression	Hypermethylated
CYP1A1 *Cytochrome P450 family 1 subfamily A member 1*	Xenobiotic metabolism and AHR-linked inflammatory signalling	Hypermethylated
RUNX3 *Runt related transcription factor 3*	Th1 differentiation and Th1/Th2 balance	Mixed/CpG-specific
TSLP *Thymic stromal lymphopoietin*	Epithelial-derived type 2 immune activation	Hypomethylated
HLA-E *Human leukocyte antigen E*	Antigen presentation and NK-cell regulation	Hypermethylated
HSP90AA1/HSPA1A/B *Heat shock proteins*	Antigen processing, chaperone function and MHC-I presentation	Hypermethylated

##### 3.1.1.1 Epigenetic regulation of type 2 immune responses

Neonatal immune function is characterised by a relative polarisation towards a type 2 immunophenotype, marked by IL-4, IL-5 and IL-13 signalling and reduced Th1 counter-regulation. Antenatal smoking exposure further amplifies this phenotype, leading to higher cord blood IL-8 and tIgE, and increased production of IL-13 and IL-5 in allergen-stimulated CBMCs (35–42). Smoke exposure leads to differential methylation of thymic stromal lymphopoietin (TSLP), an epithelial-derived cytokine that is a key initiator of type 2 responses, and increased methylation of IL-10, which has been linked to childhood asthma risk (43, 44). Smoking exposure is also associated with hypermethylation of HLA-E, HSP90AA1 and HSPA1A/B, genes involved in antigen processing and presentation, potentially influencing allergic sensitisation and downstream type 2 immune responses (45, 46). In addition, the hypermethylation of RUNX3, a key transcription factor of Th1 polarisation, may further shift the Th1/Th2 balance towards type 2 immunity (47).

##### 3.1.1.2 Epigenetic regulation of pro-inflammatory immune responses

Antenatal smoking exposure promotes a pro-inflammatory immune phenotype, characterised by increased proliferative responses and pro-inflammatory cytokine production following mitogenic stimulation (40, 41, 48–50). Cigarette smoke contains xenobiotic ligands capable of activating AhR-dependent signalling, with downstream regulation of xenobiotic metabolism and inflammatory immune responses. Consistent with this pathway, antenatal smoking exposure is associated with differential methylation of AHRR and CYP1A1, key regulators of AhR signalling and immune responses to microbial pathogens (51–58). MYO1G is hypermethylated, impacting T and B cell adhesion and migration (59, 60). The downregulation of IL-10 and T_reg_-specific FOXP3 enhancer (TSDR) further promotes pro-inflammatory immune responses through the suppression of their immunomodulatory function (61).

##### 3.1.1.3 Epigenetic regulation of haematopoiesis

Antenatal smoking exposure is associated with hypomethylation and upregulation of GFI1, a key regulator of haematopoiesis across both myeloid and lymphoid cell lineages (62, 63).

Smoking exposure is associated with increased maternal and cord blood miR-223, a microRNA involved in neutrophil, macrophage and Treg differentiation; in exposed infants higher miR-223 expression correlates with lower Treg numbers and increased risk of atopic dermatitis (64, 65). Together, GFI1 hypomethylation and miR-223 upregulation suggest that antenatal smoking exposure alters epigenetic regulation of haematopoiesis, reducing regulatory T-cell development and increasing susceptibility to atopic disease.

##### 3.1.1.4 E-cigarette exposure

E-cigarette use has risen considerably in recent years and is often perceived as a safer alternative to smoking, with prevalence estimates in pregnancy of around 2.8% (66). Maternal vaping has been associated with adverse neonatal outcomes, particularly low birth weight and small-for-gestational-age birth, with some studies also suggesting increased risk of preterm birth (67, 68). Human evidence linking maternal vaping to offspring immune programming remains limited. However, animal studies suggest that prenatal e-cigarette exposure can alter fetal and neonatal lung immune pathways, modify DNA methylation, and increase susceptibility to later immune dysfunction. Reported effects include altered cytokine expression, dysregulated T-cell activation pathways, exaggerated Th2-mediated allergic airway inflammation and lasting changes in antiviral immune responses, including altered macrophage, neutrophil and CD4+ T-cell responses (69–72). Notably, some effects occur after nicotine-free aerosol or third-hand e-vapour exposure, suggesting that solvents, flavourings and aerosol residues may contribute independently to immune programming (73).

#### 3.1.2 Maternal obesity

Maternal obesity is one of the most common and clinically important maternal health exposures during pregnancy. During pregnancy, maternal obesity induces a physiological state of chronic low-grade inflammation, metabolic dysregulation and hormonal imbalances, which converge to shape fetal immune development. Clinically, this is reflected in increased early neonatal morbidity, including early-onset sepsis, respiratory morbidity, hypoglycaemia and higher rates of neonatal intensive care admission (74, 75). This increased vulnerability may persist beyond the neonatal period, with cohort data linking maternal obesity to higher rates of infection-related hospital admission during infancy and childhood (76). These clinical associations are reflected in immunological studies showing altered immune-cell composition, dysregulated innate and adaptive responses, and coordinated changes in the neonatal epigenome (**Table 3**) (77, 78).

**Table 3:** The impact of maternal obesity on neonatal immune function.

Characteristic	Impact		Ref
Cord Blood Immune Cell Frequencies	↑T_C_ ↑T_H_ ↑Treg ↑NKT ↓Eϕ ↓Mϕ ↓pDCs ↓total myeloid cells		(243–247)
Cytokine Profile	↑CRP ↑TNF-α ↑IL-6 ↑IL-8 ↑IL-12p40 ↑IFN-α2 ↑Leptin ↓IL-10 ↓Adiponectin		(91, 92, 247–252)
Immunoglobulin Profile	↑tIgM		(253)
Cytokine Response in Stimulated CBMCs	PHA stimulated -	- ↑IL-1β ↑IL-6 ↑TNF-α	(249)
T-Cell Function	Anti-CD3/CD28 stimulation	- ↓IL-4 ↓Polyfunctional IL-2+ IL-4+ T cells	(89)
Monocyte Function	↓mRNA IL-1β, IL-10 and IL-12β TLR2/1 agonist stimulation LPS Stimulation E. coli stimulation	- ↑IL-6 ↑TNF-α - ↓IL-1α ↓IL-1β ↓IL-1RA ↓IL-10 ↓CXCL1 ↓CCL4 ↓CCL5 ↓CXCL10 - ↓IL-6 ↓IL-12p70 ↓IL-10 ↓PD-L1 ↓CCL4 ↓CCL11 ↓CXCL11	(245, 254)
Dendritic Cell Function	TLR1/2 stimulation - LPS stimulation -	- ↑IL-6 ↑TNF-α - ↑TNF-α	(254)
Macrophage Function	↑TNF-α, IL-4R and IL-10 Expression M1 stimulation (IFN-γ + LPS)	- ↑TNF-α RNA ↑IL-1β RNA	(255)
Markers of Oxidative stress	↓Superoxide Dismutase Activity		(252)

T_C_, Cytotoxic T cell; T_H_, Helper T cell; T_R_, Regulatory T cell; NKT, natural killer T cell; Eϕ, Eosinophil; Mϕ, Monocyte; pDC, plasmacytoid dendritic cell; CRP, C-reactive protein; TNF-α, Tumour Necrosis Factor alpha; IL, interleukin; IFN, interferon; tIgM, total immunoglobulin M; PHA, Phytohaemagglutinin; TLR, Toll-like receptor; LPS, lipopolysaccharide; CXCL, CXC motif Chemokine ligand; CCL, chemokine ligand.

↓, decreased; ↑, increased.

##### 3.1.2.1 Metabolic reprogramming of the neonatal immune system

Maternal obesity leads to differential methylation in at least 38 genes with direct or indirect roles in immune function. Although many of these genes are classically metabolic, particularly those involved in fatty-acid metabolism and AMPK-mediated catabolic signalling, these pathways are central to immune-cell activation and fate (79). In this context, altered methylation of metabolic genes may reshape immune responsiveness by disrupting the metabolic reprogramming required after activation to support rapid proliferation and clonal expansion (79, 80).

Maternal obesity alters cord blood adipocytokine levels with increased leptin and reduced adiponectin. Leptin functions as an immune-metabolic cytokine, enhancing both innate and adaptive immune activation through JAK-STAT3 and PI3K-mTOR signalling pathways (81). Leptin also provides a mechanism for epigenetic crosstalk through the recruitment of histone acetyltransferases (HAT) via JAK/STAT, thereby enabling potential histone acetylation at immune promoters (82). The epigenetic sensitivity of this pathway is supported by a randomised controlled trial in which a Mediterranean diet during pregnancy led to hypermethylation of the leptin gene locus, with a corresponding reduction in cord blood leptin (83).

##### 3.1.2.2 Epigenetic regulation of immune-cell activation and lineage differentiation

In CBMCs, there are several differentially methylated genes involved in PI3K-AKT-mTOR signalling, which regulates immune-cell activation and metabolic adaptation, and Wnt signalling, which controls immune-cell differentiation and tissue homeostasis. There is also increased expression of miR-516b-5p, miR-520a-3p, miR-1323 and miR-525-5p, which regulate both adaptive and innate immune-cell proliferation and differentiation (84–88).

The elevated CD4+ T cell frequencies seen following maternal obesity correlate with the hypomethylation of several key genes involved in T-cell activation including CD3 and CD28. The impaired activation of stimulated Th cells correlates with co-ordinated changes in DNA methylation and chromatin accessibility in genes with roles in T-cell signalling, VDJ recombination, activation and differentiation (89). T-cell activation and differentiation is further impacted by the downregulation of miR-221 and miR-155 (85). There is hypomethylation of the RUNX3 and NFATC2 loci, usually associated with memory function, with hypermethylation of naïve-maintaining regulator FOXP1, consistent with an epigenetic shift from naïve to a memory-like CD4+ T cell profile (89).

The impaired monocyte immune responses are reflected in the epigenome with downregulation of genes involved in antigen processing and presentation, and myeloid cell differentiation (90).

##### 3.1.2.3 Epigenetic regulation of cytokine production and inflammatory signalling

The impaired cytokine responses following mitogenic stimulation are partially mediated by differential methylation in genes responsible for modulation of TGF-β, IL-1, IL-2, IL-4, IL-5 and IL-6 production (84, 91). There is also decreased expression of miR-221 and miR-155, both of which enhance pro-inflammatory cytokine production (92–94). Downstream cytokine signalling pathways are also affected, with hypomethylation of the cytokine-response transcription factor STAT3 (95). The dysregulated TLR responses correlate with differential methylation of genes involved in innate immune sensing, NF-κB signalling, and cytokine production, including ALPK1 and PTEN, which regulate pattern-recognition receptor activation, and PI3K-AKT-mTOR-dependent cytokine responses (88).

##### 3.1.2.4 Gestational weight gain and the epigenetic regulation of immune function

Independent of pre-pregnancy obesity, gestational weight gain (GWG) can impact offspring immune function. GWG leads to increased levels of inflammatory cytokine expression with levels of IL-1β and IL-6 rising in correlation with GWG. This finding was most prominent in those with a low pre-pregnancy BMI (96). Greater GWG was associated with differential methylation of genes within canonical inflammatory gene networks, including increased methylation of NFKB1 and MMP7 (97).

#### 3.1.3 Alcohol

Although the developmental harms of pregnancy alcohol exposure are well established, including impaired fetal growth, preterm birth, fetal alcohol spectrum disorders and a higher incidence of neonatal sepsis and atopic disorders, its effects on neonatal immune programming remain poorly defined (98–102). The reported impact of alcohol exposure on lymphocyte numbers is inconsistent in human studies with contradictory results, but suggestive of suppressed T and B cell numbers, alongside elevated eosinophils and tIgE (98, 99). In vitro exposure of lymphocytes to ethanol results in reduced NK activity (100). Animal studies have highlighted differences in cytokine expression in the blood, prefrontal cortex and hippocampus (101, 103). The mechanism by which alcohol exposure affects neonatal immune function is not fully understood, but may relate to dysregulation of the hypothalamic–pituitary–adrenal axis (104). Epigenome–wide association studies have not identified any differentially methylated genes associated with antenatal alcohol exposure, and direct evidence linking alcohol exposure to epigenetic regulation of neonatal immune function remains limited (105, 106).

#### 3.1.4 Mental health

Maternal depression is associated with reduced IL-10 production in stimulated CBMCs (107). Similarly, high levels of maternal stress also alter CBMC responses, with increased IL-8, IL-13, TNF-α and reduced IFN-γ following stimulation (108).

The impact of maternal mental health and psychosocial stress on epigenetics has been extensively investigated. The majority of differentially methylated genes identified relate primarily to nervous system development and DNA binding (109). Some, including PRKCZ and CYBA, have an indirect role in immune function, promoting NF-κB activation or supporting generation of reactive oxygen species, respectively (110, 111). Pharmacological treatment for depression leads to enrichment in pathways responsible for MHC class II antigen presentation and leukocyte migration (109, 112).

### 3.2 Environmental exposure

#### 3.2.1 Pollution

Antenatal exposure to air pollution is a well-established risk factor for adverse fetal and neonatal outcomes, including growth restriction, low birth weight, preterm birth and neonatal respiratory morbidity (113–115). At a cellular level, particulate matter and combustion-derived pollutants can induce oxidative stress, xenobiotic-response signalling and innate immune activation, providing plausible upstream routes to downstream epigenetic and immune effects (116, 117). Compared with maternal smoking, however, pollution exposure results in fewer and smaller magnitude changes to the infant methylome, likely reflecting a lower cumulative or more heterogeneous exposure (55, 118–120). Despite this, multiple studies have identified a subtler but significant impact on the immuno-epigenome, including alterations to histone modification networks, cytokine regulation and immune-cell composition (**Table 4**).

**Table 4:** The impact of pollution on neonatal immune function.

Characteristic	Exposure type	Timing of exposure	Impact	Ref
Cord Blood Immune Cell Frequencies	PM2.5	Whole pregnancy 1^st^ trimester 2^nd^ trimester 3^rd^ Trimester Last 15 days	↓pDCs ↑T_H_ ↓NK ↓B ↓T_H_1 ↓T_H_ ↑NK ↑B ↓T_C_ ↓T_H_1	(121, 256, 257)
	PM10	Whole pregnancy 1^st^ trimester 2^nd^ trimester 3^rd^ Trimester Last 15 days	↓T_R_ ↑T cell ↑T_H_ ↑CCR6-ve T_H_1 ↓T_R_ ↑CCR6 -ve T_H_1 ↑T_H_2 ↓T_R_ ↑T_H_ ↓T_R_ ↓T_R_	(256)
	NO_2_	Whole pregnancy 1^st^ trimester 2^nd^ Trimester Last 15 days	↓NK cell† ↓T cells ↓T_C_† ↑CD161+ve T_H_17 ↑T cells ↑T_H_ ↑NK	(169, 256)
Cytokine Profile	PM2.5	Whole pregnancy	↑IL-10 ↓IL-27	(131, 258)
	PM10	Whole Pregnancy 3^rd^ Trimester Last 3 days	↑IL-10 ↑IL-1β ↓IL-10	(258, 259)
	NO_2_	Whole Pregnancy	↑IL-1β ↑IL-6 ↑IL-10 ↑TSLP* ↑IL-33*	(125, 258)
Immunoglobulin Profile	PM2.5	Early Gestation Late Gestation	↓cIgE ↑cIgE	(122)

*Only in female infants, † Contradictory findings between studies.

pDC, plasmacytoid dendritic cells; T_H_, Helper T cell; NK, natural killer; B, B cell; T_C_, Cytotoxic T cell; IL, interleukin; TSLP, thymic stromal lymphopoietin; cIgE, cord blood immunoglobulin E; PM, particulate matter; NO_2_, nitrogen dioxide

↓, decreased; ↑, increased.

##### 3.2.1.1 Temporal and sex-specific effects

Pollution-associated immune and epigenetic effects vary by time of exposure. Alterations in immune cell frequencies and cord blood immunoglobulin profiles vary depending on the period of pregnancy in which exposure predominantly occurs, with both first and third trimester exposure having the strongest associations (121, 122). This is reflected by temporal changes in both DNA methylation and the expression of regulatory microRNAs (miR-20 and miR-21), which modulate immune responses and self-tolerance (123, 124).

The impact of pollution exposure on cytokine expression is dependent on infant sex, with increased TSLP and IL-33 only evident in female infants (125), with corresponding sex-specific changes in IGF2 and H19 methylation (126).

##### 3.2.1.2 Epigenetic regulation of cytokine production

Pollution exposure is associated with methylation changes in several genes regulating cytokine responses. Higher PM2.5 and carbon monoxide are associated with increased IL-4 methylation, while NO_2_ exposure resulted in increased IL-10 methylation (127). PM1, PM2.5 and PM10 were all associated with LINE-1 hypomethylation, which can increase retrotransposon activity and activate innate immune sensing pathways, including cGAS-STING and type-I interferon signalling (128–130). High PM2.5 exposure throughout pregnancy, but most significantly in the third trimester, led to maternal chromatin remodelling, with coordinated changes in histone post-translational modifications in maternal immune cells and was subsequently associated with low neonatal IL-27 (131).

##### 3.2.1.3 Epigenetic regulation of immune-cell differentiation

Pollution exposure also affects several pathways involved in immune-cell differentiation. Second-trimester PM2.5 exposure was associated with differential methylation and increased miR-17 and miR-222 expression, both of which can influence PI3K-AKT/mTOR signalling, a pathway central to T- and B-cell proliferation (132, 133). Antenatal proximity to green space is associated with differential methylation of HLA-DRB5 and RPTOR (134), the latter a core mTOR-complex component involved in immune-cell activation and differentiation (135, 136). PM10 exposure is associated with FOXP3 and NOTCH4 hypomethylation, implicating altered regulation of Treg differentiation and broader T-cell fate determination (119, 120, 127).

#### 3.2.2 Heavy metal exposure

Heavy metals, including arsenic, mercury, cadmium, copper, lead and chromium, also represent a significant component of the fetal exposome. They are of particular concern in low- and middle-income countries with rapid urbanisation and industrialisation, leading to soil and groundwater contamination (137). Even at very low levels of exposure, they can have diverse systemic effects with prenatal and early-life exposures linked to immune dysregulation and an increased incidence of immune-mediated disorders including atopic dermatitis, food allergy, pre-school wheeze and allergic rhinitis (138, 139). These effects are mediated through multiple mechanisms, including disruption of redox homeostasis, altered DNA methyltransferase activity and modulation of immune-signalling pathways, resulting in long-term alterations in immune-cell differentiation, signalling and immune tolerance (**Table 5**).

**Table 5:** The impact of heavy metal exposure on neonatal immune function.

Characteristic	Metal	Impact	Ref
Cord Blood Immune Cell Frequencies	Arsenic	↓T_H_ ↑T_H_2: ↑T_C_ ↑T_H_2:T_H_1 ratio ↑Eϕ ↑Mast cells	(140, 141, 260, 261)
	Methylmercury	↓Naïve T_H_	(149)
	Mercury	↑NKT ↑B cell ↑naïve T_H_ ↓Mϕ	(151, 152)
Cytokine Profile	Arsenic	↑IL-1β ↑IL-8 ↑IFN-γ ↑TNF-α	(140, 262, 263)
	Barium	1^st^ Trimester Exposure - ↑IL-8 ↑TNF-α ↑IL-17	(148)
	Chromium	↑IL-13	(264)
	Cadmium	↑MIP-1α 1^st^ Trimester Exposure - ↑IL-8 ↑TNF-α ↑IL-17	(148, 265)
	Copper	1^st^ Trimester Exposure - ↑IL-8 ↑TNF-α ↑IL-17	(148)
	Lead	↑IL-13	(264)
	Mercury	↑IL-10 ↓IL-6	(148)
	Molybdenum	↑MCP-3 ↑MIP-1α	(265)
	Nickel	↑MCP-3	(265)
	Vanadium	1^st^ Trimester Exposure - ↑IL-8 ↑TNF-α ↑IL-17	(148)
	Zinc	↑MCP-3 ↑MIP-1α	(265)
Immunoglobulin Profile	Mercury	↑cIgG ↑Autoantibodies	(153)
T Cell Function	Mercury	↓Proliferative response ↓T cell activation markers	(149)
Cytokine Response in Stimulated CBMCs	Mercury	PHA stimulation - ↓TNF-α ↓IL-10*	(150)

*Potential confounding differences between groups.

↓T_H_, Helper T cell; ↑T_C_, Cytotoxic T cell; Eϕ, Eosinophil; NKT, Natural Killer T cell. IL, interleukin; IFN, interferon; TNF-α, tumour necrosis factor alpha; MIP-1α, Macrophage inflammatory Protein-1 alpha; MCP-3, Monocyte chemotactic Protein 3; Ig, immunoglobulin; PHA, Phytohaemagglutinin

##### 3.2.2.1 Arsenic and epigenetic regulation of immune signalling

Antenatal arsenic exposure is characterised by increased oxidative stress, inflammation and polarisation towards a type 2 immunophenotype in exposed neonates (140, 141). Transcriptomic analyses of exposed infants show a shift towards immune-cell tissue infiltration with increased expression of genes related to chemotaxis and cell adhesion (141). There is enrichment in NF-κB and IL-1β-centred gene networks suggestive of heightened innate immune and inflammatory signalling (142). This is mirrored in epigenome studies with over-representation of immune-signalling pathways (MAPK-ERK, TREM1, clathrin-mediated endocytosis, TLR and NF-κB) (143–145). Collectively, these transcriptomic and epigenomic shifts align with an exaggerated Th2-polarised pro-inflammatory immune phenotype seen in exposed infants.

##### 3.2.2.2 Arsenic and epigenetic regulation of inflammation

Arsenic exposure has a significant impact on the neonatal epigenome, with global DNA hypomethylation and variable expression of miRNAs, with sex-specific methylation patterns, boys being more affected than girls (143, 144, 146).

There is hypomethylation in several pro-inflammatory genes, including IL1B and COX2, with evidence of persistence throughout childhood (147). Prenatal arsenic exposure was associated with increased expression of multiple miRNAs involved in immune function, including inflammatory signalling (let-7a, miR-16, miR-26b, miR-107), interferon/TLR–NF-κB pathways (miR-20a, miR-20b), immune-cell activation and proliferation (miR-17, miR-195), chemotaxis and endocytosis (miR-126), and Th2/allergic regulation (miR-98) (143). Together, the changes in DNA methylation and miRNA expression converge to downregulate TLR/IFN signalling and amplify pro-inflammatory responses, providing a mechanistic link to altered neonatal immune function and future allergic risk.

##### 3.2.2.3 Mercury and impaired cellular immunity

*In utero* mercury exposure impacts the immune system both in terms of composition and function, with alterations in immune cell frequencies, suppression of T-cell activation and proliferation, and cytokine production (148–152). Increased expression of cord blood IgG and autoantibodies also imply increased autoreactivity (153). These alterations in immune function coincide with changes in the neonatal epigenome with exposure-associated methylation changes in PON1 (oxidative stress regulation); MED31 and GGH (gene transcription and immune signalling), suggesting impaired cellular immunity and dysregulated humoral responses driven by oxidative and epigenetic reprogramming (154–156).

##### 3.2.2.4 Cadmium and immune regulatory pathways

Cadmium exposure is associated with widespread methylation at loci regulating immune-cell activation and differentiation, innate immune responses, cell adhesion and migration, immune signalling and cytokine production, though many of these changes appear transient and do not persist throughout childhood (157). Exposure demonstrates both sex-specific and time-point-specific effects with exposure during early pregnancy producing a distinct methylation profile compared with later exposure (158, 159).

##### 3.2.2.5 Other heavy metals, antigen presentation and immune signalling

Several other heavy metals exert an epigenomic effect on immune function. Copper, chromium, caesium, mercury, magnesium and manganese are all associated with differential methylation of human leukocyte antigen (HLA), potentially altering antigen presentation and immune tolerance (160). Lead exposure was also linked to differential methylation in genes involved in immune-cell adhesion and trafficking (161). In populations with mixed heavy metal exposure from e-waste, transcriptomic analyses showed enrichment of several pathways including NF-κB, TGF-β and apoptotic signalling pathways (162).

##### 3.2.2.6 Lead and LINE-1 methylation

Lead exposure is associated with reduced LINE-1 methylation, a marker of broader epigenomic instability that has been implicated in immune dysregulation, autoimmunity and cancer immune responses (128, 129, 163, 164). Lead exposure was also linked to differential methylation in genes involved in immune-cell adhesion, supporting a potential link to altered intercellular communication and inflammatory signalling (161).

#### 3.2.3 Volatile organic compounds

Volatile organic compounds (VOCs), including benzene, toluene, ethylbenzene and xylene, contribute to indoor air pollution (165). They can originate from industrial processes; however, within the home, the dominant sources include smoking, cooking and cleaning products (166, 167). They have been implicated in a range of health disorders and are strongly associated with increased risk of childhood asthma (168). However, their impact on neonatal immune function is not well characterised.

In cord blood, maternal benzene exposure has been associated with a reduced number of CD4+CD25+ T cells, used as a proxy for Tregs, without significant changes in other lymphocyte subsets. The other VOCs appear to have no effect on cord blood immune cell frequencies (169). VOC exposure was associated with an increase in IL-4 expressing Th2 cells and a reduction in IFN-γ-expressing Th1 cells (170). Collectively, these findings suggest a polarisation to dysregulated type 2 immune responses.

In adults, toluene exposure was associated with reduced miR-155 expression which can polarise towards a Th2 phenotype (65, 171). Both benzene and toluene exposure were associated with increased miR-223 which may contribute to altered myeloid regulation and enhanced Th2-associated inflammation observed in allergic disease, although miR-223 can also serve anti-inflammatory roles depending on context (65). In exposed adults, occupational VOC exposure is associated with significant changes in the methylome (172), however, there are currently no studies exploring the impact of maternal VOC exposure on the offspring methylome.

#### 3.2.4 Farm exposure

Maternal farm exposure has been consistently linked to a reduced incidence of type 2-mediated immune disorders in offspring (173). Infants of exposed mothers show increased Treg and FOXP3 expression with reduced lymphoproliferative responses, suggesting enhanced immune regulation. These findings correlate with the hypomethylation of the FOXP3 gene locus, seen following antenatal milk-farm exposure, suggesting epigenetic imprinting of tolerance (174).

Maternal farm exposure during pregnancy also modulates Th cytokine balance, generally favouring a pro-inflammatory Th1-type response over Th2 polarisation. In infants of exposed mothers, stimulated CBMCs produced significantly higher IFN-γ, IL-6 and TNF-α than non-exposed infants (175). In contrast, in farm-exposed neonates, IL-5 production is suppressed following mitogen or allergenic stimulation (174).

Maternal farm exposure also results in lower cord blood IgE, which is classically associated with allergic sensitisation (23). Maternal dairy-farm exposure leads to increased levels of B-cell activation factor (BAFF), a B-cell maturation cytokine associated with a reduced risk of allergy (176).

Although beyond the scope of this review, postnatal exposures and their influence on the formation of the neonatal microbiome play a vital role in shaping postnatal immune trajectories (177). These postnatal factors likely act in tandem with prenatal programming and collectively shape long-term risk of allergic and atopic disease.

### 3.3 Perinatal factors

#### 3.3.1 Mode of delivery

Mode of delivery plays a key role in shaping the neonatal immune system following birth (**Table 6**), with caesarean section (CS) associated with an increased risk of asthma, atopic dermatitis, allergic rhinitis and allergic disease throughout childhood (178–181).

**Table 6:** The Impact of vaginal delivery on immune function – compared to caesarean section.

Characteristic	Impact		Ref
Cord Blood Immune Cell Frequencies	↑Total leukocyte count ↑Nϕ ↑NK ↑Mono ↓B cells ↓Total T cell count ↑T_C_ ↓T_H_ ↑Treg		(266–271)
Cytokine Profile	↑IL-2 ↑IL-6 ↑IL-8 ↑IL-18 ↓IL-13 ↓IFN-γ ↓TNF-α ↓TGF-β ↓GCSF ↓GM-CSF ↓TNF-α mRNA ↓IFN-γ mRNA		(189–191, 272–276)
Immunoglobulin Profile	↑IgA ↑IgG		(277–279)
Cytokine Response in Stimulated CBMCs	**Mitogenic ** ConA/PMA Stimulation PHA Stimulation LPS Stimulation TLR1/2 Stimulation CpG Stimulation RSV Stimulation **Allergenic ** HDM antigen stimulation Cat Dander Stimulation	-↑IFN-γ -↑IFN-γ and IL-12 -↑IFN-γ ↑IL-12 ↓IL-8 -↑IL-6 ↑TNF-α -↓IL-12p40 -↑IL-12p40 ↑IL-13 ↑IFN-γ	(275, 280–283)
Neutrophil Function	↓Spontaneous apoptosis ↑Chemotaxis response ↑Integrin alpha expression ↑E. coli stimulation-induced ROS ↑CD11b expression ↑IL-8 receptor A expression		(284–286)
Monocyte Function	↑TLR2/4 expression ↑TLR2/4 mRNA		(287)
Regulatory T Cells	↓FOXP3 expression		(271)

Nϕ, Neutrophil; NK, Natural killer cell; Mono, Monocyte; T_C_, Cytotoxic T cell; T_H_, Helper T cell; Treg, regulatory T cell; IL, Interleukin; IFN, Interferon; TNF, Tumour Necrosis Factor; TGF, Transforming growth factor; GCSF, Granulocyte colony stimulating factor; GM-CSF, Granulocyte macrophage colony stimulating factor; ConA, Concanavalin; PMA, Phorbol myristate acetate; PHA, Phytohaemagglutinin; LPS, lipopolysaccharide; TLR, Toll-like receptor; RSV, respiratory syncytial virus; HDM, House dust mite.

Bold text indicates the type of stimulation used in stimulated CBMC experiments: mitogenic stimulation or allergenic stimulation.

↓, decreased; ↑, increased.

The effect of delivery mode on neonatal immune function should be considered within the context of the wider immune transition that occurs around birth. The latter stages of pregnancy are characterised by the gradual loss of immune tolerance, immune-cell infiltration of the myometrium and upregulation of IL-1, IL-6 and TNF-α (182–185). The process of labour itself represents a major physiological stressor both for the mother and fetus, collectively contributing to dynamic changes in neonatal immune function (186).

Beyond mode of delivery, other perinatal factors can impact neonatal immune function. Reduced cord blood pH, a marker for perinatal stress, was associated with reduced lymphocyte numbers (187). In preterm infants, a longer duration of labour was associated with increased neutrophil antigen expression (188). Compared with an elective CS, emergency CS resulted in higher levels of IL-1β, IL-6, TNF-α, IL-18 and MCP-1 (189–191).

Mode of delivery results in detectable changes in the epigenome with CS resulting in hypermethylation of the ELA2 gene, a neutrophil granule enzyme, and IRF1, interferon regulatory factor 1 (192). There is also pathway enrichment for genes in TGF-β signalling and phagocytosis pathways (193).

## 4 Maternal microbiome as an exposome-epigenome interface

The maternal microbiome is emerging as an important interface between environmental exposures and the developing fetal immuno-epigenome. Although early literature suggested that the *in utero* environment had a defined microbiome, more recent robust studies that better controlled for potential contamination have shown no consistent evidence of a resident fetal or placental microbiome (194–196). As a result, vertical transmission of the maternal microbiota is likely restricted to birth and the postnatal period, and though this plays a vital role in shaping the neonatal microbiome and subsequently postnatal immune trajectories, this pathway remains beyond the scope of this review (177, 197, 198).

During gestation, maternal microbial communities modulate fetal immune development through the transplacental transfer of circulating metabolites (177). Maternal microbiome composition and its functional metabolic outputs may be shaped by several components of the fetal exposome, including diet, obesity, antibiotic exposure and broader environmental exposures (177, 199). Their impact on fetal immune development likely relies less on microbial composition alone and more on which circulating bacterial metabolites can reach, or signal across the maternal-fetal interface (200, 201).

The strongest mechanistic evidence relates to short-chain fatty acids (SCFAs), including acetate, propionate and butyrate, produced through microbial fermentation of dietary fibre. SCFAs can signal through free fatty acid receptors, including FFAR2 and FFAR3, but their relevance to epigenetic regulation lies in their ability to inhibit histone deacetylases (202, 203). This can increase histone acetylation, alter chromatin accessibility and regulate pathways involved in immune tolerance and inflammatory responses (203). Experimental and animal studies show that SCFAs can promote regulatory T-cell differentiation and modify allergic airway inflammation; however, direct human evidence linking maternal SCFAs to fetal immune-cell epigenetic remodelling remains limited (204, 205).

Beyond SCFAs, microbiota-derived tryptophan metabolites may influence immune programming through AhR-dependent signalling. Microbial effects on retinoid, bile acid and one-carbon metabolism provide additional potential links to epigenetic regulation, although these pathways are less clearly defined in human fetal immune development (177, 206).

## 5 Immune compartments

**Figure 3:**
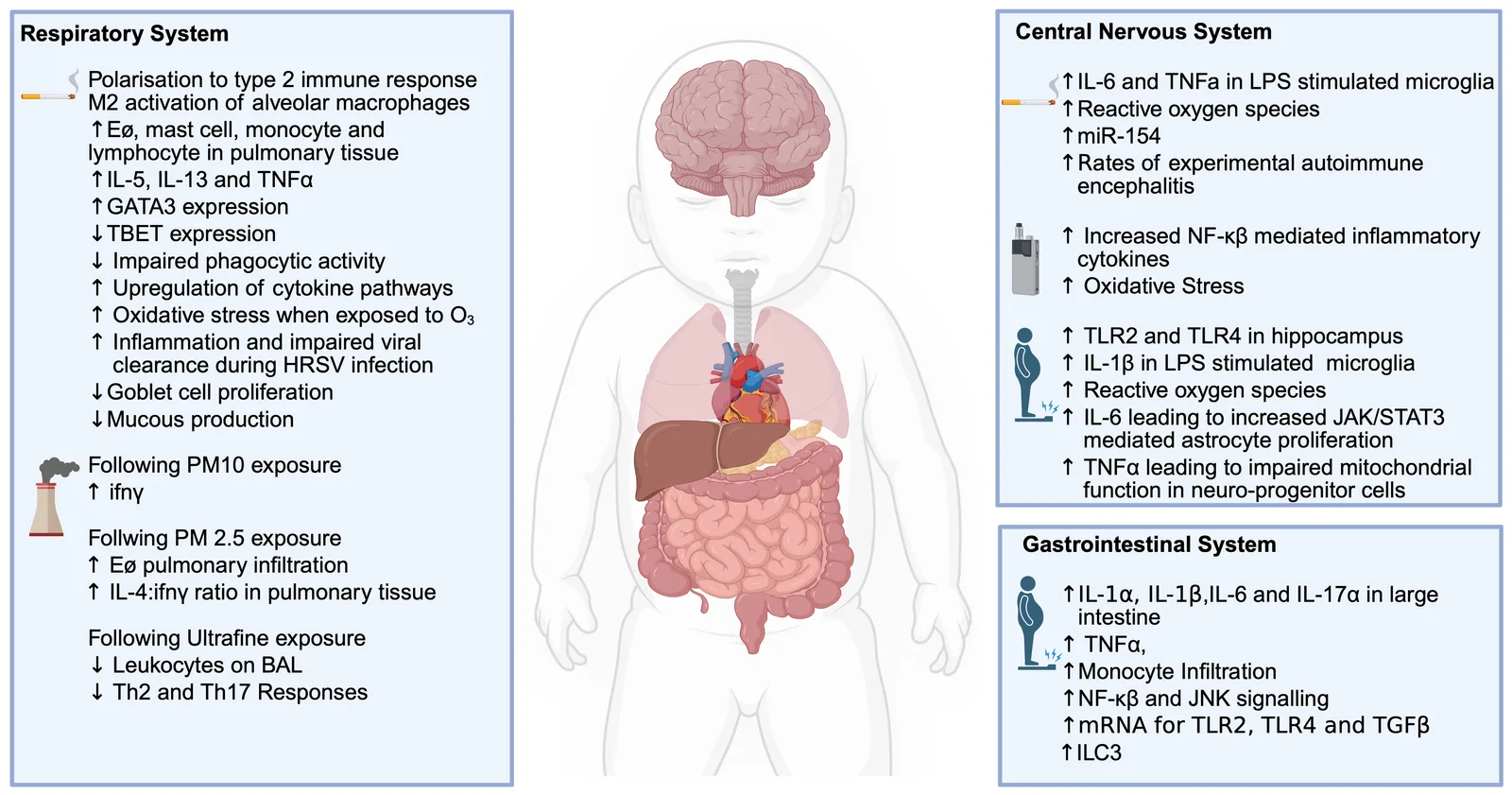
Compartment-specific immune effects of antenatal exposures. Tissue-specific immune alterations reported in animal models following antenatal exposures. Findings are summarised across the respiratory, gastrointestinal and central nervous systems and may not directly reflect human neonatal immunity. Respiratory system references (207–221):; central nervous system references (222–226):; gastrointestinal system (227, 228):. Created in BioRender. Dodwell, W (2026). https://BioRender.com/onljwmx.

Cohort studies involving infants rely on cord or peripheral blood samples to ascertain the impact of a given risk factor on immune function. However, the immune system is highly compartmentalised and therefore blood samples may only give a partial picture of the overall impact on the immune system, and direct sampling of lung and gut tissues is rarely feasible or ethical. This is particularly important for the respiratory and gastrointestinal tracts, which represent major interfaces between the developing immune system and the external environment, and are key sites for microbial, dietary and inhalational antigen exposure after birth. Animal models therefore provide important complementary evidence by allowing direct assessment of tissue-specific immune effects following controlled antenatal exposures. **Figure 3** highlights the impact of antenatal exposures on the central nervous, respiratory and gastrointestinal systems. These findings are inferred from animal models and may not fully reflect human compartment immunity.

## 6 Discussion

In this review, we describe the fetal exposome’s crucial role in the development of the neonatal immune system. Through epigenetic mechanisms, maternal health and environmental exposures during fetal life can have a lifelong impact on gene expression with lasting consequences on immune function and the susceptibility to immune-mediated disease. Taken together, these findings suggest that the biological effects of maternal and environmental exposures are not uniform across pregnancy but depend on the developmental window in which exposure occurs. This supports the concept of sensitive periods during fetal immune programming, particularly in early gestation when epigenomic plasticity is greatest.

Evidence across diverse exposures, including maternal smoking, obesity, pollution and chemical exposures, shows a converging pattern of immune dysregulation, most notably a polarisation towards a type 2 immunophenotype, impaired regulatory function, and dysregulated innate immune responses. These findings suggest that fetal exposures may shape the balance between immune tolerance, inflammatory regulation and host defence during early life, but the functional and clinical significance of these changes remains poorly understood. In particular, few studies have linked exposure-associated immune alterations to functional immune responses, infection susceptibility, vaccine responses, allergic sensitisation or later immune-mediated disease.

Current studies often consider risk factors in isolation, potentially underestimating the cumulative or confounding effects of co-exposures, particularly in the context of social deprivation, where multiple risk factors often converge. Despite consistent associations with adverse early-life health outcomes, including increased infant morbidity and mortality, the influence of social deprivation on the epigenetic regulation of immune function remains poorly examined.

The complexity of fetal immune development cannot be captured by studying isolated outcomes or exposures. Real-world exposures overlap and interact, particularly in the context of social deprivation.

Addressing this uncertainty will require study designs that connect exposure, mechanism and immune phenotype with long-term immune outcomes within the same individual. Future research should move beyond single-exposure models and adopt a multi-omics, systems-level approach that integrates fetal exposures, epigenetic, transcriptomic, metabolomic and immunological analyses across key biological compartments.

Longitudinal studies linking early-life multi-omic alterations to immune-mediated disorders and infectious disease will be essential for identifying biomarkers of risk and resilience. Ultimately, this knowledge must inform the development of antenatal and early-life strategies that can reduce disease burden, narrow health inequalities, and support lasting immune health.
